# A rolled-up-based fabrication method of 3D helical microrobots

**DOI:** 10.3389/frobt.2022.1063987

**Published:** 2022-11-29

**Authors:** Zihan Wang, Xueliang Mu, Liyuan Tan, Yukun Zhong, U. Kei Cheang

**Affiliations:** ^1^ Department of Mechanical and Energy Engineering, Southern University of Science and Technology, Shenzhen, China; ^2^ Department of Biomedical Engineering, University of Groningen and University Medical Center Groningen, Groningen, Netherlands; ^3^ Department of Mechanical and Manufacturing Engineering, University of Calgary, Calgary, AB, Canada; ^4^ Shenzhen Key Laboratory of Biomimetic Robotics and Intelligent Systems, Southern University of Science and Technology, Shenzhen, China; ^5^ Guangdong Provincial Key Laboratory of Human-Augmentation and Rehabilitation Robotics in Universities, Southern University of Science and Technology, Shenzhen, China

**Keywords:** rolled-up technology, swelling effect, helical microrobots, controllable, feedback control strategy

## Abstract

While the potential of using helical microrobots for biomedical applications, such as cargo transport, drug delivery, and micromanipulation, had been demonstrated, the viability to use them for practical applications is hindered by the cost, speed, and repeatability of current fabrication techniques. Hence, this paper introduces a simple, low-cost, high-throughput manufacturing process for single nickel layer helical microrobots with consistent dimensions. Photolithography and electron-beam (e-beam) evaporation were used to fabricate 2D parallelogram patterns that were sequentially rolled up into helical microstructures through the swelling effect of a photoresist sacrificial layer. Helical parameters were controlled by adjusting the geometric parameters of parallelogram patterns. To validate the fabrication process and characterize the microrobots’ mobility, we characterized the structures and surface morphology of the microrobots using a scanning electron microscope and tested their steerability using feedback control, respectively. Finally, we conducted a benchmark comparison to demonstrate that the fabrication method can produce helical microrobots with swimming properties comparable to previously reported microrobots.

## 1 Introduction

Micro/nanorobots can access hard-to-reach places and target specific locations in the body (Mhanna et al., 2014; Qiu et al., 2015; Felfoul et al., 2016; Chen et al., 2017). It is conceivable that these tiny machines will soon play important roles in the biological and medical fields, especially in tissue engineering and targeted therapy. There are many types of micro/nanorobots driven by different power sources, such as chemical fuel (Ma et al., 2015; Zhou et al., 2019), electric field (Loget and Kuhn, 2011; Kim et al., 2014), light (Palagi et al., 2016; Shahsavan et al., 2020), ultrasound (Li et al., 2015; Aghakhani et al., 2020), or magnetic field (Yu et al., 2018). Among these power sources, magnetic fields can safely penetrate biological barriers and other materials (Chen et al., 2018). Consequently, magnetic actuation seems to be a very promising avenue to actuate micro/nanorobots, especially when operating *in vivo*.

Inspired by *Escherichia coli* swim using rotating flagella (Berg and Anderson, 1973), magnetic helical micro/nanorobots had been proposed and manufactured using various methods, including self-scrolling (Zhang et al., 2009a), glancing angle deposition (GLAD) (Ghosh and Fischer, 2009), direct laser writing (DLW) (Tottori et al., 2012; Medina-Sánchez et al., 2016), template-assisted electrodeposition (TAE) (Zeeshan et al., 2014), and bio-templating synthesis (BTS) (Gao et al., 2014). In recent years, the focus is gradually shifting to the demonstration of their manipulation and functions, such as cargo transport (Tottori et al., 2012; Huang et al., 2014). However, there is a lack of development in trying to streamline the manufacturing process to massively produce helical microrobots with consistent structures, controllable helical parameters, and high repeatability. Most of the aforementioned methods can produce helical microrobots with excellent swimming properties, but their deployment and widespread usage in practical applications might be hindered by high cost, complicated fabrication processes, low throughput, or inconsistent geometries. To find manufacturing methods that can satisfy the requirements for large number deployment, parallel fabrication methods must be considered. Conventional parallel fabrication technologies at the microscale generally produce 2D patterns; this becomes an issue as the helices are 3D structures. Therefore, the stress-induced rolled-up technology based on photolithography has gradually gained importance for the fabrication of 3D microstructures (Zhang et al., 2009b; Mei et al., 2011; Smith et al., 2011; Sun et al., 2014; Tian et al., 2017; Tian et al., 2018; Xu et al., 2018; Xu et al., 2019).

To generate the stress within the 2D structures, the traditional rolled-up technology often involved complex fabrication procedures and expensive equipment. For instance, a molecular beam epitaxy machine was used and a dual-layer of semiconductor-metal materials was deposited when using the self-scrolling method to fabricate helical microrobots (Zhang et al., 2009a). In another example, expensive equipment for glancing angle deposition (GLAD) was used to produce anisotropic stress within 2D microstructures (Li et al., 2012a). In this paper, we propose a method to fabricate helical microrobots using a roll-up process achieved using conventional and commonly available microfabrication technologies: photolithography and electron beam evaporation. This method utilizes the roll-up of the photoresist to bend outer the metal layer, forming magnetic helical microrobots after the removal of the photoresist. The swimming properties of the helical microrobots in this work are comparable to the previously reported helical microrobots, which validates the feasibility to use the proposed fabrication method. Since the fabrication method only requires the use of conventional microfabrication technologies, this work has the potential to be useful for creating a variety of 3D complex microstructures with ease and may serve as a foundation for fabricating helical microrobots for large-number deployments in future applications. In short, the proposed fabrication method not only reduces the complicity of the current rolled-up technology but also reduces the cost of fabrication.

## 2 Materials and methods

**FIGURE 1:**
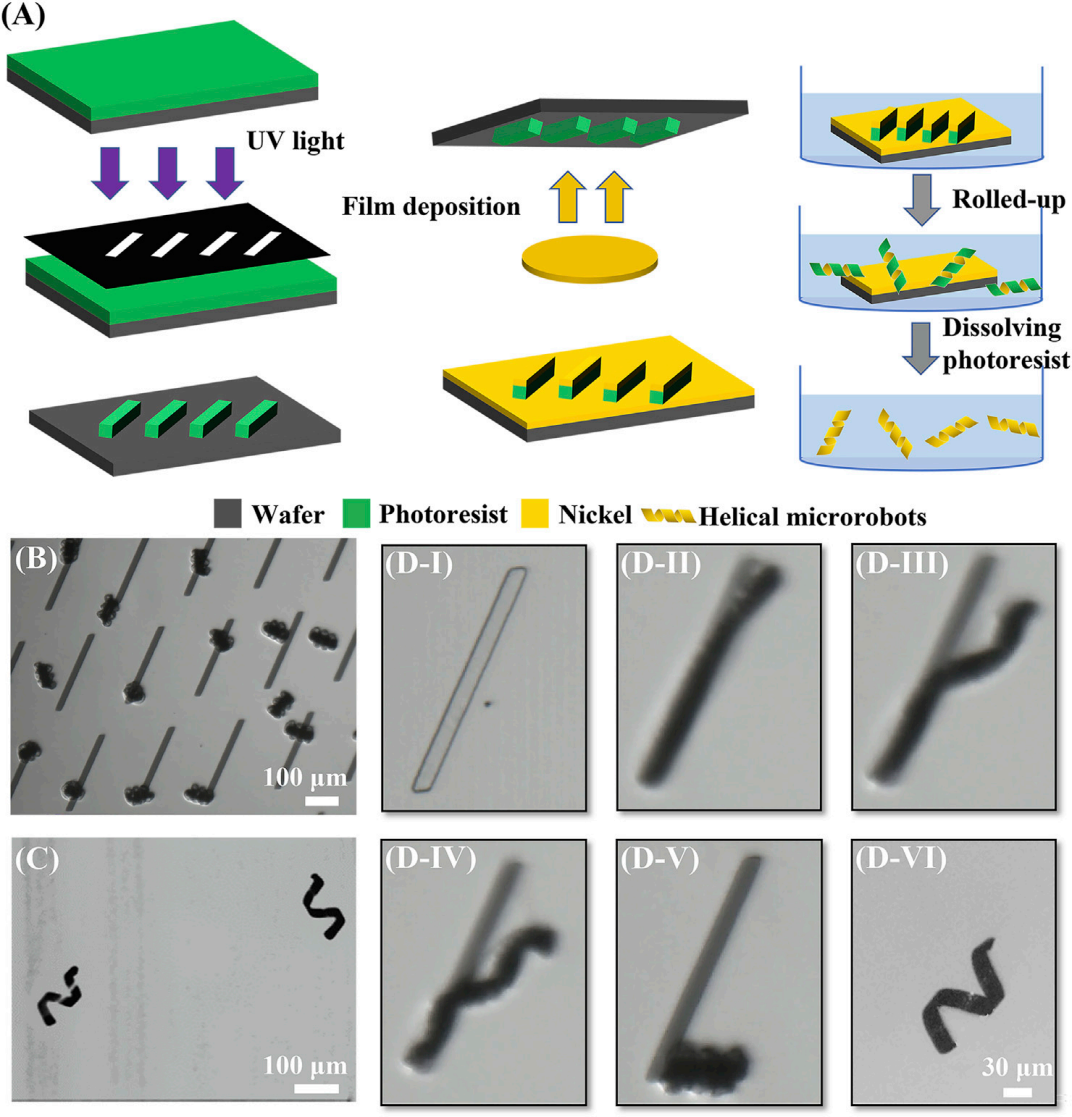
Fabrication procedure of helical microrobots. **(A)** Schematic diagram of the fabrication procedure. **(B)** Image of coils after rolling. **(C)** Image of helical microrobots *via* an optical microscope. **(D)** Microscope images of the rolled-up process. **(D–I)** 2D pattern after physical vapor deposition; **(D-II)** beginning of the rolled-up process when photoresist began to swell and one tip began to lift; **(D-III)** rolling up along the long edge and the other tip began to roll up; **(D-IV)** the two tips rolled up from opposing sides and met at the point of strongest interfacial adhesion; **(D-V)** rolled up into a coil as swelling completes; **(D-VI)** coil loosened into helical microrobot after the removal of photoresist sacrificial material.

The fabrication procedure is shown in Figure 1A. First, photoresist (MicroChemicals, AZ® nLof 2070) was spin-coated at 3000 rpm on a clean silicon wafer. The photoresist is then made into a sacrificial layer through photolithography (SUSS, MA6); parallelogram/rectangular shapes patterned on a mask were transferred to this photoresist layer. Then, a 100 nm nickel layer was deposited on the 2D photoresist patterns *via* electron beam evaporation (HVV, TF500); the nickel will make up the entire body of the microrobots. Next, the sample was immersed in N-Methyl Pyrrolidone (NMP) solution (Aladdin, 99.5%); subsequently, the photoresist was in contact with the NMP solution and swelled up causing the 2D patterns to spontaneously roll up into the coils. The final 3D helical-shaped nickel microstructures emerged when all the photoresists dissolved away in the NMP solution. Finally, the sample was washed three times using isopropanol (Aladdin, 99.5%) and then placed in deionized water for experiments later. The coils (before photoresist dissolution) and final helical microrobots (after photoresist removal) could be observed in Figures 1B,C, respectively.

## 3 Results

### 3.1 Mechanism analysis

To elucidate the mechanism of the rolled-up process caused by the swelling of the photoresist layer, the entire process was recorded at 100 frames per second for observation (see Supplementary Video S1). The entire rolled-up process using a parallelogram pattern with a length of 300 μm and a tilt angle of 60° is shown in the zoomed-in snapshots of Supplementary Video S1 in Figure 1(D-I)–1 (D-V). First, the NMP solution was added to the 2D parallelogram patterns. After the photoresist absorbed solvent molecules from the NMP solution, the macromolecular polymers would begin to swell and roll up. Due to the edge effect (Alben et al., 2011) and the internal stress generated by the swelling at the interface between the photoresist and the nickel layer, one tip of the 2D pattern was lifted off; subsequently, the 2D pattern rolled up along the direction parallel to the short edge, as shown in Figure 1(D-II). Next, the other tip lifted off in the direction perpendicular to the respective short edge, and the pattern began to roll up from the other tip, as shown in Figure 1(D-III). The different etch rates would produce an anisotropic driving force; thus, the two tips would have different lifting directions and rollup from opposite directions; this caused the two opposing tips to form two independent microhelices. Then, the two sides met at the point of strongest interfacial adhesion causing the two microhelices to combine into one single microhelix, as shown in Figure 1(D-IV). When the roll-up process was finished, the pattern became a coil, as shown in Figure 1(D-V). The cross-linked photoresist, which was immersed in the 80°C NMP solution, continued to swell until it reached maximum volume, at which point the polymer network structure broke down and slowly dissolved in the NMP solution. The photoresist sacrificial material took about two days to be completely removed, leaving only the nickel layer. Without the constraint from the photoresist sacrificial material, the coil was loosened into a helix with a single layer of nickel, as shown in Figure 1(D-VI); this final structure is representative of the magnetic helical microrobots.

According to Bell et al, (2006), if the orientation of a nanomembrane strip deviates from the rolling direction, a helical structure can be formed. In our experiments, we observed that the photoresist rolled perpendicular to the short side, which is in agreement with the experimental observation and theoretical results of Chun et al, (2010). The orientation of the photoresist in a parallelogram pattern deviated from the rolling direction; thus, the parallelogram patterns are prone to roll up into helical structures after swelling.

During wet etching, the photoresist absorbed the NMP molecules and swelled. The swelling of the photoresist induced strain in the metal layer, which caused the structures to roll up into coils. The outer layer photoresist of the coils would dissolve eventually, and the metal layer was left to constitute the helical microrobots. Therefore, the microrobots are comprised of a single layer of nickel. A mathematical model (Li et al., 2012b) is included to relate the radius of the rolled-up structures with the swelling of the photoresist. The radius *R* of the rolled-up structure is given by the following equation:

$$R=\frac{d_{1}+{d_{2}}^{\prime }\left\{3{\left(1+\frac{d_{1}}{{d_{2}}^{\prime }}\right)}^{2}+\left(1+\frac{d_{1}}{{d_{2}}^{\prime }}∙\frac{Y_{1}}{Y_{2}}\right)\left[{\left(\frac{d_{1}}{{d_{2}}^{\prime }}\right)}^{2}+{\left(\frac{d_{1}}{{d_{2}}^{\prime }}∙\frac{Y_{1}}{Y_{2}}\right)}^{-1}\right]\right\}}{6\varepsilon^{\prime }{\left(1+\frac{d_{1}}{{d_{2}}^{\prime }}\right)}^{2}}$$ (1)

where $d_{1}$ and ${d_{2}}^{\prime }$ represent the thickness of the metal layer and the swollen photoresist respectively, $Y_{1}$ and $Y_{2}$ are Young’s moduli of the metal layer and photoresist respectively, and $\varepsilon^{\prime }$ is the nominal strain along the rolling direction. The nominal strain $\varepsilon^{\prime }$ consist of the strain *C* of the metal layer during the deposition and the strain ${\varepsilon_{p}}^{\prime }$ of the swollen photoresist. Thus, it can be expressed as:

$$\varepsilon^{\prime }={\varepsilon_{p}}^{\prime }+C.$$ (2)

**FIGURE 2:**
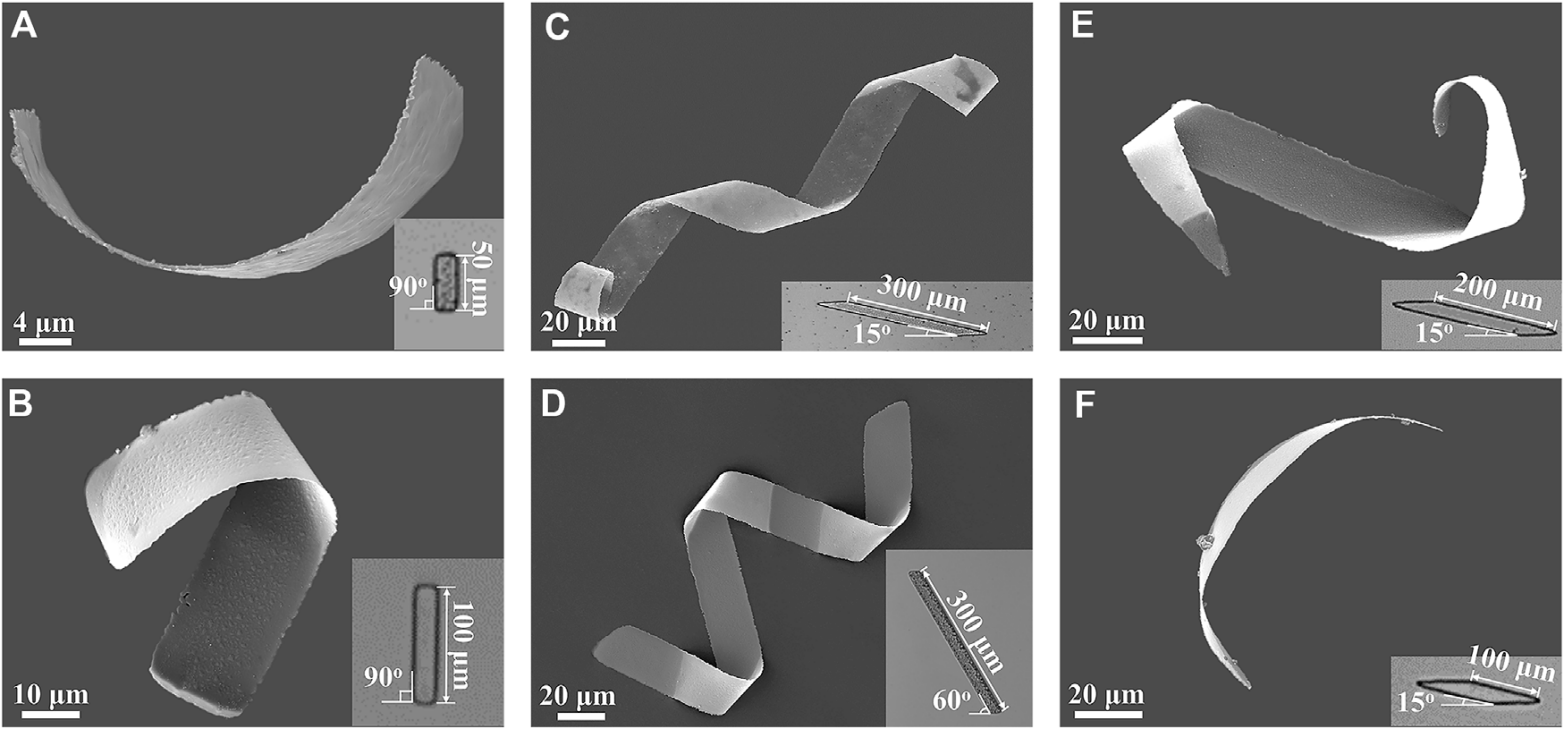
SEM images of helical microrobots fabricated using rectangular and parallelogram templates; the insets are the microscopy images of the corresponding 2D patterns. Rectangular templates with lengths of **(A)** 50 μm and **(B)** 100 μm. Parallelogram templates with the length of 300 μm and tilt angles of **(C)** 15° and **(D)** 60°. Parallelogram templates with the length of **(E)** 200 μm and **(F)** 100 μm and tilt angles of 15°.

The geometry and dimension of the 2D patterns used in experiments were varied to examine the effects of the 2D geometrical parameters on the shape of the helical microrobots. Two kinds of patterns were examined - rectangular and parallelogram patterns. Each of these patterns rolled up into different types of structures according to the way the photoresist swelled. As shown in the scanning electron microscope (SEM) images in Figures 2A,B, helical microrobots were obtained through rectangular templates with lengths of 50 μm and 100 μm. However, the helical angles of the helical microrobots created with these lengths were random. This could be attributed to the change in the folding direction, which is determined by the lowest bending energy state (Chun et al., 2010). To further improve the controllability of the helical parameters of the microrobots during the manufacturing process, parallelogram patterns with tilt angles of 15° and 60° were used. Each tilt angle was observed to have a different folding direction causing the parallelogram patterns to roll up into microhelices with different helical angles. For instance, a 2D parallelogram with a tilt angle of 60° will produce a helical structure with a helical angle of 58 ± 4° (*n* = 20, the detailed dimensions of 20 helical microrobots are summarized in Supplementary Table S1; these helical microrobots are obtained from 4 batches). A 2D parallelogram with a tilt angle of 15° will produce a helical structure with a helical angle of 36 ± 4° (*n* = 20, the detailed dimensions of 20 helical microrobots are summarized in Supplementary Table S2; these helical microrobots are obtained from 4 batches). Representative helical microrobots with helical angles of 36°, and 57° were fabricated using the aforementioned respective tilt angles, and their corresponding SEM images are shown in Figures 2C,D.

### 3.2 Size scalability

The number of turns of the helical microrobots, which also determines their length, can be controlled by tuning the length of the 2D patterns. For rectangular patterns that exceed the length of 100 μm, helical microstructures would not be formed, and delamination between the nickel and sacrificial layer will occur. The delamination is due to a larger folding curvature when the rectangular patterns with a high aspect ratio roll up from the short edge. Microrobots created using parallelogram patterns, on the other hand, do not have this problem. To determine the relationship between the number of turns of the helical microrobots and the length of 2D parallelogram patterns, parallelogram templates with lengths of 100 µm, 200 µm, and 300 µm and a tilt angle of 15° were tested. Using parallelogram templates with lengths of 300, 200, and 100 um, representative helical microrobots with 2, 1, and 0.5 turns, respectively, were created, as shown in Figures 2C,E, and Figure 2F; this indicates that the number of helical turns can be affected by the length of the parallelogram patterns. Note that the number of turns obtained from 300 μm patterns is very consistent; it is only when we decreased the length to 100 and 200 μm did we see inconsistency in the number of turns. This is because the strain ${\varepsilon_{p}}^{\prime }$ caused by the swollen photoresist varies with its length, leading to unpredictable rolling directions. With the unpredictable rolling direction, while we could conclude that the number of turns will increase with the length of the parallelogram patterns, we could not quantitatively describe the relationship between the length of the 2D patterns and the number of turns.

### 3.3 Swimming test

**FIGURE 3:**
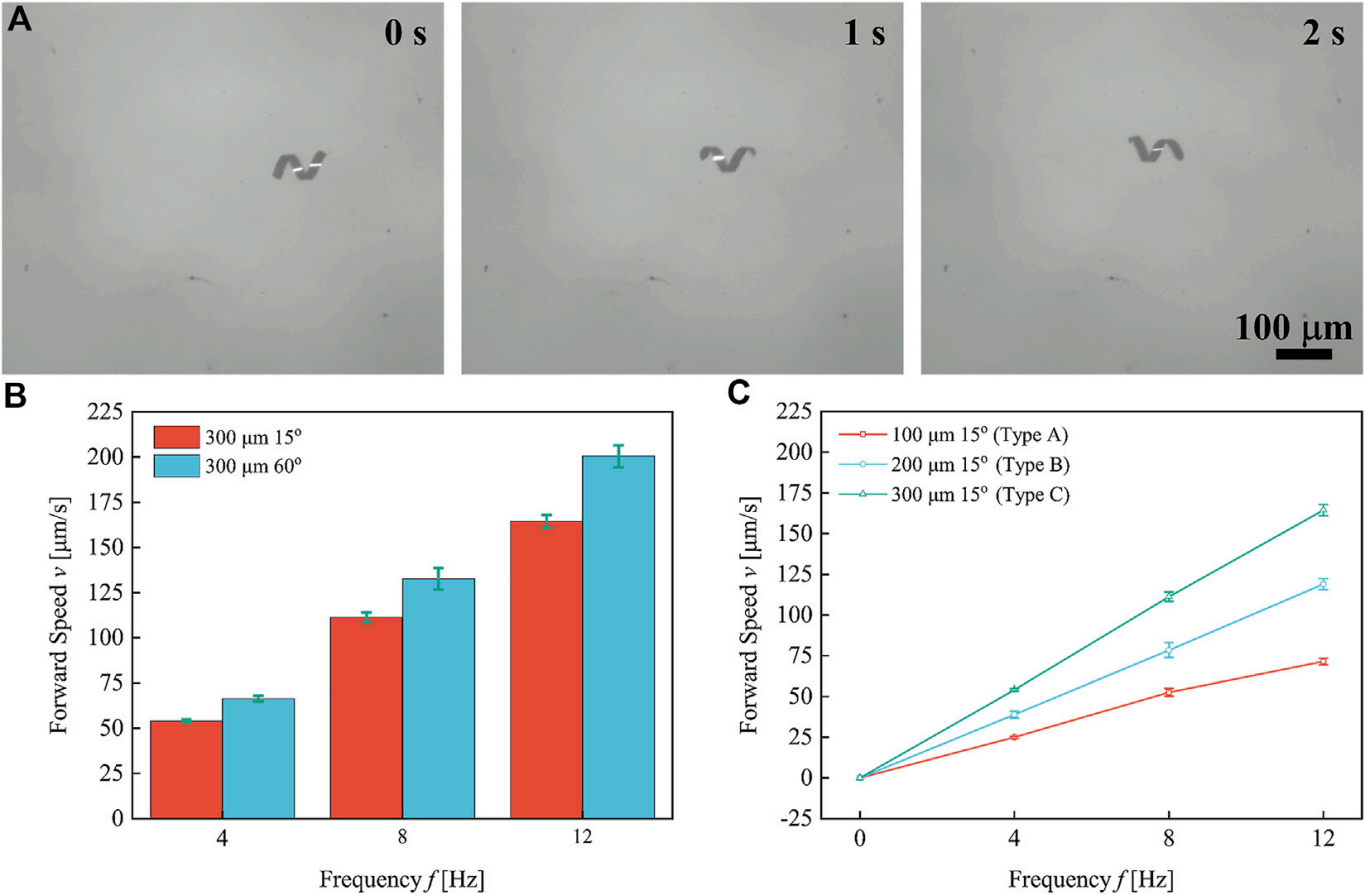
Forward swimming speed of helical microrobots under different rotational frequencies. **(A)** The time-sequence swimming of the helical microrobot fabricated through the parallelogram templates with the length of 300 μm and tilt angles of 60° under the rotational frequency of 4 Hz. **(B)** The forward swimming speed of helical microrobots with different helical angles *versus* frequency. **(C)** The forward swimming speed of helical microrobots with different body lengths *versus* frequency.

The swimming properties of helical microrobots with different helical parameters were tested in deionized water under the actuation of a rotating magnetic field (RMF). The detailed swimming test procedures and the mechanism of the magnetic coil system can be found in the Supplementary Material S1. Figure 3A shows the time-sequence swimming of the helical microrobot fabricated through the parallelogram templates with the length of 300 μm and tilt angles of 60° under the rotational frequency of 4 Hz. The forward swimming speed of the helical microrobots is plotted against rotational frequency, as shown in Figures 3B,C. Each data point represents the average speed of five microrobots and the respective error bars represent the standard deviation. The results indicate a linear relationship between forward swimming speed and rotational frequency.

The speed of the helical microrobots as a function of geometric parameters and rotational frequency is expressed as (Tottori et al., 2012)

$$v=\frac{\left(\xi_{n}-\xi_{II}\right)\sin\theta \cos\theta }{2\left(\xi_{n}\sin^{2}\theta +\xi_{II}\cos^{2}\theta \right)}Df,$$ (3)

where $\xi_{n}$ and $\xi_{II}$ are drag coefficients perpendicular and parallel to the helical axis, θ is the helical angle, D is the diameter of helical microrobots, and f is the rotational frequency of the external field. As shown in Figure 3B, the helical microrobot fabricated using parallelogram templates with a length of 300 μm and a tilt angle of 60° exhibited higher swimming speed owing to the larger diameter; this agrees with Eq. 3. Helical microrobots with a different number of turns were tested for their swimming speed, as shown in Figure 3C. Three types of helical microrobots were created using parallelogram templates with lengths at 100 μm, 200 μm, and 300 μm and a tilt angle of 15°; they are designated as Type A, Type B, and Type C microrobots, respectively. The helical microrobots in Figures 2C,E,F are representative of the microrobots used in this test. As shown in Figure 3B, the Type C microrobots swam the fastest.

The comparison of swimming ability between our helical microrobots and the previously reported helical microrobots can be made using dimensionless speed

$${\tilde{U}}_{\max}=U_{\max}/Lf,$$ (4)

where ${\tilde{U}}_{\max}$ is the maximum dimensionless speed, $U_{\max}$ is the maximum speed, and L is the corresponding body length of helical microrobots (Pak et al., 2011). The below compares the dimensionless speed, calculated using Eq. 4, of previously reported helical microrobots that were prepared using different fabrication methods. In terms of structure and shape, the microrobots manufactured by the self-scrolling process are classified as binormal helical microrobots with rectangular cross-sections, while the microrobots that were manufactured by the DLW and BTS processes are classified as normal helical microrobots with circular cross-sections (Morozov and Leshansky, 2014). The diameter-to-length ratios of the microrobots produced by these four methods are 0.068, 0.247, 0.284, and 0.326 respectively. The two normal helical microrobots fabricated by DLW and BTS have similar diameter-to-length ratios; thus, their maximum dimensionless speeds, 85.7 and 87.1 respectively, are similar. The microrobots fabricated using the swelling mechanism have the largest diameter-to-length ratio; thus, they have a higher maximum dimensionless speed. The helical nanorobots that were fabricated using the GLAD method (Ghosh and Fischer, 2009) are 2 μm in length and 256 nm in diameter, can reach 40 μm/s at 150 Hz, and have a maximum dimensionless speed of approximately 133. Although the diameter-to-length ratio of the helical nanorobots is calculated to be 0.128, they exhibit the highest dimensionless speed; this is because they were made up of hard magnetic materials (cobalt), magnetized transversally, and fabricated as normal helices with helical angles of 35°–45° (Morozov and Leshansky, 2014). The favorable comparison validates the swimming performance of the magnetic helical microrobots fabricated using the swelling mechanism.

### 3.4 Steering

**FIGURE 4:**
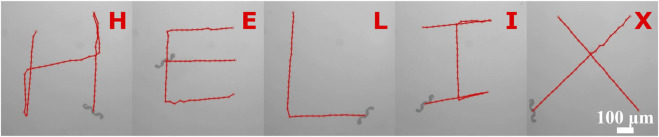
The trajectory of a helical microrobot following pre-programmed tracks.

To verify the steerability of a helical microrobot, a microrobot was steered to swim along pre-programmed tracks to write out the word HELIX (see Figure 4 and Supplementary Video S2). While the microrobots are swimming close to the substrate, the hydrodynamic interaction with the surface causes drifting motion that leads to a deviation from the intended swimming direction (Peyer et al., 2010). To account for this, a feedback control strategy was applied to the microrobot’s motion control to compensate for the deviation from the intended path (Cheang et al., 2017). The implementation method is described in the Supplementary Material.

## 4 Discussion and conclusion

The helical microrobots were created from rolled-up parallelogram structures triggered through the swelling of the photoresist sacrificial layer. When the polymer structure of the photoresist broke down, the dissolution of the photoresist would happen in the NMP solution. Ultimately, helical microrobots with a layer of nickel were acquired. The results show the helical angle of the helical microrobots can be tuned by adjusting the angle of the parallelogram patterns and the number of turns will increase proportionately with the length of the parallelogram templates. These findings will provide insights into the fabrication of helical microrobots with a variety of geometry parameters and offer experimental validations for previous theoretical results on the effects of different helical parameters to swimming performance.

Compared with the self-scrolling method, our fabrication method does not require multiple-layer deposition, and it can be realized in four steps using commonly available equipment. For GLAD, DLW, and TAE, a specialized deposition machine or a high-performance laser lithography system is required, which may hinder basic research on helical microrobots due to the availability of equipment. Using two-photon polymerization (TPP) methods (including DLW and TAE), each robot must be created one by one. Thus, research work using TTP to create helical microrobots might be hindered by time and cost. While TTP has obvious advantages in terms of material and precision control over geometrical features, the fabrication method in this work can present an alternative means to obtain helical microrobots using a parallel fabrication process with the advantages of low cost, high throughput, and equipment availability. For BTS, natural helical biotemplates can be acquired from various sources such as spiral xylem vessel plant fibers, helical microorganisms (such as Spirulina), and lotus-root fibers. Biotemplates need to be carefully prepared to avoid damage and have geometrical variations among structures. Although the researchers did not represent quantitative data to verify the consistency of the helical microrobots fabricated *via* this method, control over the helical parameters of the biotemplates can be challenging due to the mechanical isolation processes and ductile properties of the biological materials. In the case of the flagellar biotemplated microrobots, for instance, flagella obtained from live bacteria vary in length and are easily deformable during the biotemplating process, leading to variations in the helical structures (Ali et al., 2017). Thus, the popularity of this method is limited by either inconsistent structures or low geometry controllability. On the contrary, our helical microrobots consist of a nickel layer, and they can keep the intact helical shape during the fabrication process. Therefore, our proposed method may bring about two positive contributions: (1) the low resource investment may promote basic research on helical microrobots, and (2) the low cost and high throughput technique may be useful in the future for applications that requires a large number microrobots.

Under a RMF, the swimming speed of the helical microrobots increased linearly with the rotating frequency was proportional to its body length and diameter; this is consistent with the theoretical formula. Our proposed method can fabricate helical microrobots with low cost and high throughput while maintaining comparable swimming performance with previously reported helical microrobots. Furthermore, the steerability of the helical microrobots was demonstrated by swimming along a pre-programmed trajectory through feedback control; this also serves as a demonstration that these helical microrobots are feasible for applications that require precise control.

While the helical microrobots presented in this work were not made from fully biocompatible materials, our work successfully created a low-cost helical microrobotic platform that can be fabricated easily using widely available materials and equipment. We envision that future work by our group or others will be able to use this platform to create biocompatible microrobots by replacing/covering the nickel with biocompatible materials. For instance, it has been demonstrated that the biocompatibility of helical microrobots can be improved by coating with Titanium (Tottori et al., 2012; Gao et al., 2014) or co-depositing iron and platinum followed by one single annealing step (Kadiri et al., 2020). Thus, we believe that replacing nickel with iron or covering the nickel with platinum or titanium can make the roll-up helical microrobots biocompatible.

In summary, this work proposes a novel microfabrication method based on rolled-up technology that is more convenient and feasible for the parallel fabrication of helical microrobots for large number deployment. The helical microrobots were created from rolled-up parallelogram structures triggered through the swelling of the photoresist sacrificial layer. Our approach can allow for the manufacture of helical microrobots with different helical angles and numbers of turns. These findings will enhance the understanding of how to fabricate helical microrobots with a variety of geometry parameters using a roll-up mechanism and provide further experimental validation of the existing theoretical model for helical microrobots with different geometrical parameters. Under a RMF, the swimming speed of the helical microrobots will increase linearly with rotating frequency and was proportional to their body length and diameter. Our proposed method can fabricate helical microrobots with low cost and high throughput while maintaining swimming performance. Furthermore, the steerability of the helical microrobots was demonstrated by swimming along a pre-programmed trajectory through feedback control, demonstrating that the helical microrobots created in this work can potentially be used as a microrobotic platform for applications that require high-precision motion control, such as targeted therapy.

## Data Availability

The raw data supporting the conclusion of this article will be made available by the authors, without undue reservation.
