# Supplementary material for: A rolled-up-based fabrication method of 3D helical microrobots
Source: Front Robot AI. 2022 Nov 29;9:1063987. doi: 10.3389/frobt.2022.1063987 (PMC9744796; doi:10.3389/frobt.2022.1063987)
Supplement: Supplementary file 1 [file Table1.docx]

Supplementary Material

## Supplementary Figures and Table

### Magnetic Actuation and Microscopy Observation

The system for magnetic actuation and microscope observation equipment is depicted in Supplementary Figure 1. A 3D Helmholtz coil system is used to generate a rotating magnetic field (RMF) for magnetic actuation. Each coil pair of Helmholtz coil system is individually powered by a power supply (Kepco, BOP20-5M) with a maximum output current of 2 A. The maximum magnetic strength produced in the central area is approximately 15 mT. A Data Acquisition (DAQ) controller (National Instrument, PCI-6259) can connect the LabVIEW interface with the power supplies and realize digital-to-analog conversion. The frequency and strength of the magnetic field generated by the coils can be adjusted through the LabVIEW interface. The generated magnetic field is described as

$\left[ \begin{matrix} \boldsymbol{B}_{\boldsymbol{x}} \\ \boldsymbol{B}_{\boldsymbol{y}} \\ \boldsymbol{B}_{\boldsymbol{z}} \end{matrix} \right]\boldsymbol{=B}\left[ \begin{matrix} \left( \mathbf{sin}\boldsymbol{(\alpha)}\mathbf{cos}\boldsymbol{(\omega t)-}\mathbf{cos}\boldsymbol{(\alpha)}\mathbf{sin}\boldsymbol{(\beta)}\mathbf{sin}\boldsymbol{(\omega t)} \right)\boldsymbol{+(}\sin\left( \boldsymbol{\alpha} \right)\sin\left( \boldsymbol{\omega t} \right)\boldsymbol{+}\mathbf{cos}\boldsymbol{(\alpha)}\mathbf{sin}\boldsymbol{(\beta)}\mathbf{cos}\boldsymbol{(\omega t))} \\ \left( \cos\left( \boldsymbol{\alpha} \right)\cos\left( \boldsymbol{\omega t} \right)\boldsymbol{+}\sin\left( \boldsymbol{\alpha} \right)\sin\left( \boldsymbol{\beta} \right)\sin\left( \boldsymbol{\omega t} \right) \right)\boldsymbol{-(}\cos\left( \boldsymbol{\alpha} \right)\sin\left( \boldsymbol{\omega t} \right)\boldsymbol{-}\mathbf{sin}\boldsymbol{(\alpha)}\mathbf{sin}\boldsymbol{(\beta)}\mathbf{cos}\boldsymbol{(\omega t))} \\ \cos\left( \boldsymbol{\beta} \right)\sin\left( \boldsymbol{\omega t} \right)\boldsymbol{+}\cos\left( \boldsymbol{\beta} \right)\mathbf{cos}\boldsymbol{(\omega t)} \end{matrix} \right]$, S(1)


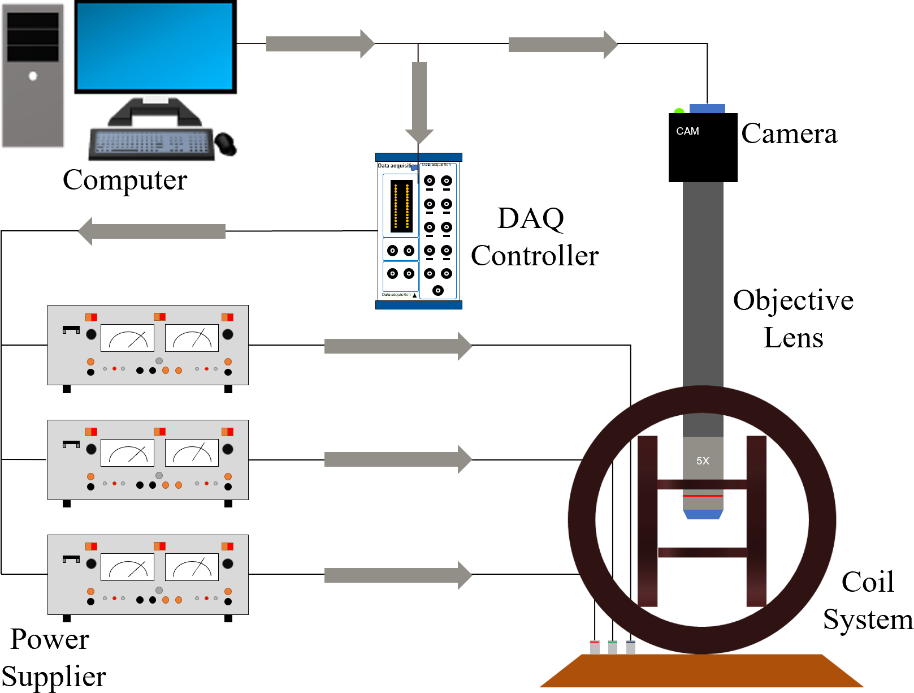


**Supplementary Figure 1.** Diagram of the system used for magnetic actuation and microscope observation.


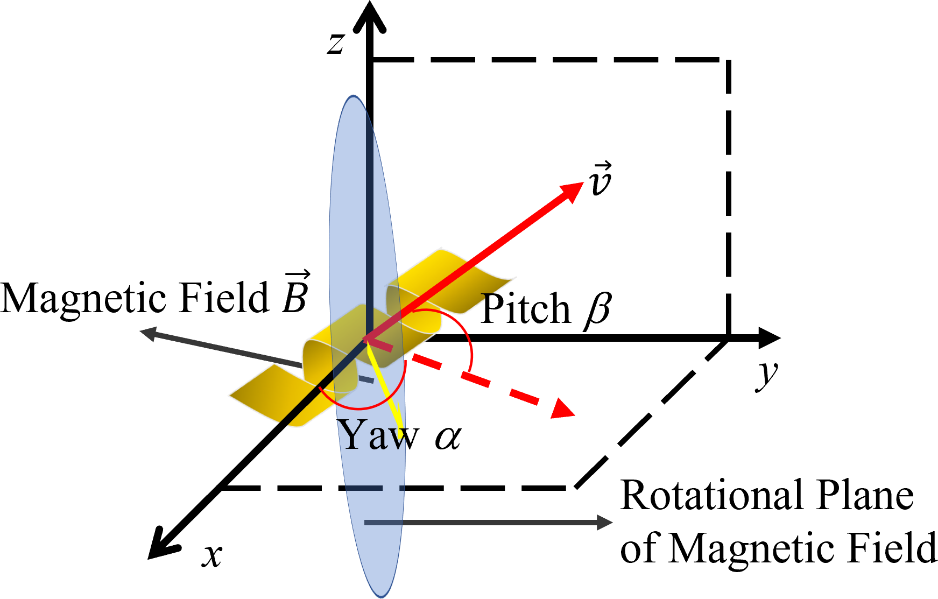


**Supplementary Figure 2.** A schematic of a microrobot and its swimming velocity relative to the rotating magnetic field.

where $B$, $\alpha$, $\beta$, $\omega$, and $t$ represent the amplitude of rotating magnetic field, the yaw of swimming direction, the pitch of swimming direction, the rotational frequency of the magnetic field, and time, respectively. For the swimming tests of the helical microrobots, the experiments were only performed on the *xy* plane. Therefore, pitch angle $\beta$ in LabVIEW platform is set to 0°. Motion control of magnetic microrobots in 3D can be achieved by setting a pitch angle greater than 0°. As shown in Supplementary Figure 2, the rotation axis (red dashed arrow) is a unit vector perpendicular to the plane of the rotation field, which can be described as follows:

$\vec{\mathbf{n}}\mathbf{=}\left[ \begin{matrix} \cos\left( \boldsymbol{\beta} \right)\mathbf{cos(}\boldsymbol{\alpha}\mathbf{)} \\ \cos\left( \boldsymbol{\beta} \right)\mathbf{sin(}\boldsymbol{\alpha}\mathbf{)} \\ \mathbf{sin(}\boldsymbol{\beta}\mathbf{)} \end{matrix} \right]$. S(2)

It is parallel to the ideal swimming direction $\vec{v}$ of helical microrobots. For imaging and video recording during experiments, a system consists of an optical microscope with a 5× objective lens (Mitutoyo, M Plan Apo), a digital camera (FLIR, BFS-U3-13Y3M-C), and the SpinView software. Videos using this system are imported into MATLAB for postprocessing and analysis.

### Swimming Test Method

The polydimethylsiloxane (PDMS) chamber with the helical microrobots was placed in the center of the coil system where the magnetic field was uniform. All the experiments to test the swimming speed of the microrobots were conducted in deionized water. According to the speed formula for helical microrobots (Morozov et al., 2017), the speed would reach the maximum value when the wobbling angle (the angle between the rotational axis and the helical axis) is 0^o^. The wobbling angle of the helical microrobots can vary with magnetic field strength. This is because the helical microrobots would adjust the wobbling angle to maintain a balanced relationship between hydrodynamic torque and magnetic torque (Cheang et al., 2014). The specific swimming test procedure is as follows. First, the magnetic field strength was tuned until the wobbling angles of the helical microrobots were 0^o^ under a rotating frequency of 12 Hz. Then, the frequency was reduced to 8 Hz and 4 Hz, while the magnetic field strength was decreased to half and one-third, respectively. Because the hydrodynamic torque was proportional to rotational frequency, the magnetic field strength was decreased accordingly without changing the wobbling angle; thus, the torque balance was maintained. As a result, the wobbling angle of helical microrobots would remain unchanged throughout the swimming test across multiple rotating frequencies.

### Feedback Control Strategy

As shown in Supplementary Figure 3, the autonomous navigation of helical microrobots was realized by a closed loop control system, which consists of path planning, motion controller, and image process algorithm. The control system was implemented in LabVIEW. First, the actual position of helical microrobots ($x_{r}\left( t \right), y_{r}\left( t \right)$) was detected through real-time image processing, and the target position ($x_{T}\left( t \right), y_{T}\left( t \right)$) was determined manually. Then, the distance $e$ and heading angle $\theta$ were calculated using the actual position and the target position. Next, a PI controller was implemented to control the heading angle and rotational frequency of helical microrobots. Finally, the magnetic coil system was controlled to generate the RMF with the corresponding rotating frequency and direction.

**
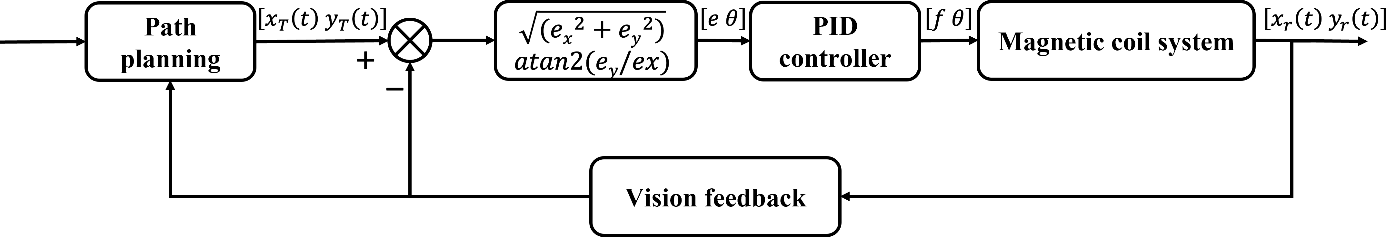
Supplementary Figure 3.** The block diagram showing the closed-loop control system for autonomous navigation along the pre-designed track.

### Supplementary Table

The geometry dimension of twenty helical microrobots fabricated through a 2D parallelogram with a tilt angle of 60° is summarized in Supplementary Table 1. These helical microrobots are obtained from four batches. The variation from one helix to another is characterized by the standard deviation in the table.

Supplementary Table 1. The geometry dimension of five helical microrobots fabricated through a 2D parallelogram with a tilt angle of 60°.

| Index | Length *L* [μm] | Diameter *D* [μm] | Helical angle *θ* [°] |
| --- | --- | --- | --- |
| 1 | 132.195 | 52.197 | 56.036 |
| 2 | 123.902 | 60.050 | 55.042 |
| 3 | 127.317 | 54.155 | 62.98 |
| 4 | 125.854 | 60.032 | 50.081 |
| 5 | 128.293 | 56.106 | 61.195 |
| 6 | 125.325 | 54.943 | 61.742 |
| 7 | 126.233 | 55.397 | 63.997 |
| 8 | 122.147 | 60.846 | 59.826 |
| 9 | 123.055 | 56.760 | 63.548 |
| 10 | 126.233 | 54.491 | 56.032 |
| 11 | 132.817 | 55.263 | 62.786 |
| 12 | 132.260 | 65.944 | 56.437 |
| 13 | 124.733 | 57.541 | 58.072 |
| 14 | 124.164 | 57.805 | 58.364 |
| 15 | 137.795 | 48.533 | 56.760 |
| 16 | 126.683 | 52.394 | 62.464 |
| 17 | 126.832 | 57.146 | 55.125 |
| 18 | 129.929 | 57.055 | 57.505 |
| 19 | 129.182 | 60.800 | 54.945 |
| 20 | 123.560 | 59.883 | 54.109 |
| mean ± SD | 127.426 ± 3.945 | 56.867 ± 3.823 | 58.352 ± 3.809 |

The geometry dimension of twenty helical microrobots fabricated through a 2D parallelogram with a tilt angle of 15° is summarized in Supplementary Table 1. These helical microrobots are obtained from four batches. The variation from one helix to another is characterized by the standard deviation in the table.

Supplementary Table 2. The geometry dimension of twenty helical microrobots fabricated through a 2D parallelogram with a tilt angle of 15°.

| Index | Length *L* [μm] | Diameter *D* [μm] | Helical angle *θ* [°] |
| --- | --- | --- | --- |
| 1 | 185.417 | 21.250 | 36.097 |
| 2 | 173.711 | 22.349 | 37.170 |
| 3 | 175.582 | 26.103 | 31.069 |
| 4 | 177.845 | 22.406 | 35.578 |
| 5 | 177.006 | 33.140 | 44.537 |
| 6 | 178.002 | 32.810 | 43.543 |
| 7 | 186.549 | 26.156 | 30.964 |
| 8 | 186.034 | 31.030 | 39.433 |
| 9 | 182.126 | 29.882 | 33.877 |
| 10 | 186.895 | 32.618 | 39.188 |
| 11 | 186.500 | 32.888 | 36.350 |
| 12 | 185.282 | 27.857 | 34.992 |
| 13 | 188.043 | 32.888 | 29.781 |
| 14 | 183.023 | 28.206 | 34.738 |
| 15 | 173.530 | 29.856 | 36.856 |
| 16 | 183.607 | 26.150 | 38.656 |
| 17 | 179.093 | 25.170 | 36.573 |
| 18 | 180.285 | 28.533 | 33.473 |
| 19 | 186.032 | 29.344 | 37.889 |
| 20 | 179.239 | 29.845 | 37.975 |
| mean ± SD | 181.690 ± 4.685 | 28.424 ± 3.728 | 36.437 ± 3.749 |

## Supplementary Videos

**Supplementary Video 1.** The rolled-up process of parallelogram templates with the length of 300 μm and the tilt angle of 15° and 60°, respectively.

**Supplementary Video 2.** Feedback control of helical microrobots along the predesigned trajectory: HELIX.

Cheang UK, Lee K, Julius AA, et al. (2014) Multiple-robot drug delivery strategy through coordinated teams of microswimmers. *Appl. Phys. Lett.* 105: 083705. doi: 10.1063/1.4893695.

Morozov KI, Mirzae Y, Kenneth O, et al. (2017) Dynamics of arbitrary shaped propellers driven by a rotating magnetic field. *Phys. Rev. Fluids* 2: 044202. doi: 10.1103/PhysRevFluids.2.044202.
